# Subacute Oral Toxicity Study of Astragalus Root Water Extract in Rats

**DOI:** 10.1155/jt/7973889

**Published:** 2025-12-17

**Authors:** Wei Du, Ping Zhang, Xiaoxian Song, Peilin He, Silan Wu, Jinping Luo, Chonggang Huang, Sixing Huang

**Affiliations:** ^1^ Chongqing Academy of Chinese Materia Medica, Chongqing, China, cqacmm.com

**Keywords:** *Astragalus membranaceus*, repeated-dose toxicity, safety profile

## Abstract

**Background:**

*Astragalus membranaceus* has a long‐standing history of use in traditional Chinese medicine (TCM). Despite its extensive historical application and the emerging scientific evidence supporting its medicinal value, the safety of its extracts, particularly concerning subchronic toxicity, remains inadequately characterized.

**Aim:**

This study conducted a subchronic toxicity assessment of Astragalus root water extract (AWE) in Sprague–Dawley rats to evaluate its safety profile.

**Methodology:**

The safety of AWE was evaluated through a 90‐day repeated‐dose toxicity study, administering a constant daily dose of 47 g of crude drug per kilogram of body weight. The animals were assessed for body weight, food consumption, rectal temperature, and rotarod performance, alongside hematological and biochemical parameters, bone marrow and immune parameters, and underwent gross necropsy and histopathological examination.

**Results:**

No treatment‐related mortality, clinical abnormalities, or gross/histopathological changes were observed. Hematological evaluations, clinical biochemical analyses, bone marrow cytology assessments, and spleen immune typing did not reveal any adverse changes. Compared to the control group, rats treated with AWE exhibited a transient decrease in food intake across five different 24‐h intervals, as well as a significant reduction in rectal temperature on Day 90. On the 90th day, the latency period on the rotating rod decreased significantly; however, during the subsequent 30‐day recovery phase, all parameters returned to baseline levels. Additionally, absolute and relative organ weights, weight gain, and fat mass remained unaffected.

**Conclusion:**

The high‐dose administration of AWE did not exhibit significant toxic effects in Sprague–Dawley rats, thereby supporting its safety within a certain dose range.

## 1. Introduction

*Astragalus membranaceus*, commonly known as “huangqi” in Chinese, has a long‐standing history of utilization in traditional Chinese medicine (TCM), where it is particularly esteemed for its ability to invigorate “Qi” and strengthen tissues. The root of *A. membranaceus* is the primary medicinal component, which has garnered significant attention due to its reported effects in alleviating fatigue, modulating the immune system, and exhibiting anti‐inflammatory properties [1, 2]. The active constituents, such as Astragaloside IV, flavonoids, and polysaccharides, are thought to play a crucial role in these therapeutic effects [3]. Nevertheless, despite its historical application and the emerging scientific evidence that supports its medicinal value, the safety profile of *A. membranaceus* extracts, especially regarding subchronic toxicity, remains insufficiently characterized, particularly in light of modern pharmacovigilance standards.

Subchronic toxicity studies are essential for elucidating the potential adverse effects of substances following repeated exposure over a substantial portion of an organism’s lifespan, typically 90 days in rodent models [4]. These studies yield critical data on target organ toxicity, systemic effects, and potential dose–response relationships, which are vital for risk assessment and the establishment of safe exposure limits in humans [5]. Given the increasing global interest in natural products and their incorporation into mainstream healthcare, it is imperative to ensure the safety of these agents through rigorous scientific evaluation.

Numerous preclinical and clinical studies have investigated the therapeutic potential of *A. membranaceus* extracts in various disease models, including cardiovascular diseases, diabetes, and immune disorders [6, 7]. However, comprehensive data on subchronic toxicity, especially in mammalian models, are notably lacking. Existing toxicological assessments typically focus on acute toxicity or limited organ‐specific evaluations, which fail to capture the nuanced effects of long‐term exposure [8, 9]. Additionally, variations in extraction methods, plant sources, and concentrations of bioactive compounds can significantly influence the pharmacological and toxicological profiles of *A. membranaceus* preparations, highlighting the need for standardized and reproducible study designs [10].

The regulatory landscape for natural health products exhibits significant variability across different jurisdictions. Some regions mandate comprehensive safety data comparable to that required for pharmaceuticals, while others depend on historical usage or traditional knowledge [11]. This regulatory diversity highlights the necessity of generating high‐quality, peer‐reviewed data to facilitate evidence‐based decision‐making and inform public health recommendations.

Currently, the primary method of consuming Astragalus root is through decoction in water [12]. In this study, we performed a thorough subchronic toxicity assessment of standardized Astragalus root water extract (AWE) in Sprague–Dawley rats, aiming to enhance our understanding of the safety profile of *A. membranaceus*.

## 2. Materials and Methods

### 2.1. Chemicals

All the solvents and reagents were of analytical grade and purchased from MACKLIN, China.

### 2.2. Plant Material and Extraction

Astragalus root samples were obtained from Professor Pengfei Tu’s team at Peking University and identified as *A. membranaceus* (Fisch.) Bge. var. *mongholicus* (Bge.) *Hsiao*. To simulate traditional clinical practices in TCM, the samples were ground into a coarse powder and subjected to repeated reflux extraction at 100°C for 1 hour each time, extracted twice, and subsequently dried to yield a yellow‐brown fine powder with a slight odor and a mildly bitter taste [13]. The extraction yield of the fine powder, calculated as (mass of extract/mass of crude drug) × 100%, is 28.5%.

### 2.3. Phytochemical Analysis of Extracts

Preliminary phytochemical analysis was conducted using the phenol‐sulfuric acid (PSA) method [14].

### 2.4. Dose Preparation

The AWE powder was dissolved in purified water at 40°C with stirring to enhance the dissolution process. The final concentration of the suspension was 4.7 g of crude drug per milliliter. Doses for the toxicological study were prepared immediately prior to administration.

### 2.5. Experimental Animals

Experimental animals Sprague–Dawley rats, weighing between 120 and 160 g, were housed in a facility maintained at a constant temperature of 22 ± 3°C and a humidity range of 40%–70%, under a 12‐h light/dark cycle. Diet and water were provided ad libitum, except during blood sample collection. Standard pellet feed was available throughout the study. The study protocol received approval from the Ethics Committee for Experimental Animals at the Chongqing Academy of Chinese Materia Medica (Approval No. YLS2022‐58) and was monitored to ensure compliance with ethical standards.

The extract was administered via gavage at the specified doses.

### 2.6. Experimental Protocol and Dose Selection

Sixty Sprague–Dawley rats were divided into two groups, with 15 animals of each sex per group. Ten animals from each group were sacrificed at the end of the dosing period (Day 91, D91), while five were sacrificed at the conclusion of the recovery period (Day 121, D121). Over a duration of 90 days, purified water and Astragalus water extract were administered daily at a dose of 47 g of crude drug per kg of body weight. Blood and organs were collected from each animal for subsequent testing and examination.

### 2.7. Measurement of Rectal Temperature in Rats

Rats were restrained, and a thermometer probe was gently inserted into the rectum for 3 min to measure rectal temperature under normal conditions (22 ± 2°C) [15].

### 2.8. Motor Behavior Analysis With a Rotarod Test

During the acclimation period, rats were pretrained on a rotarod apparatus (Beijing Zhongshi Dichuang Technology Development Co., Ltd China) at speeds ranging from 4 to 40 rpm, twice weekly prior to testing. On the test day, rats were placed on the rod at a speed of 30 rpm for 10 min, and the latency to fall was recorded to assess physical activity [16].

### 2.9. Hematology

During blood collection of a rat, approximately 2 mL of blood was withdrawn from the abdominal aorta using a syringe. The blood was kept in heparinized EDTA tubes for the measurement of hematological parameters (XT‐2000i fully automated animal hematology analyzer, Japan). These included analysis on total red blood cell count (RBC), hemoglobin (HGB), red blood cell indices (mean corpuscular volume [MCV], mean corpuscular hemoglobin [MCH], and mean corpuscular hemoglobin concentration [MCHC]), total white blood cell count (WBC), platelet count (PLT), prothrombin time (PT), and activated partial thromboplastin time (APTT).

### 2.10. Serum Biochemistry

Approximately 3 mL of blood withdrawn from each animal was extracted by centrifugation at 3000 rpm for 15 min to obtain serum. The serum collected was analyzed (AU480 fully automated biochemical analyzer, USA) for sodium, potassium, chloride, total protein (TP), albumin (ALB), total bilirubin (TBIL), creatinine (CERA), blood urea nitrogen (BUN), total cholesterol (CHOL), triglycerides (TG), blood glucose (GLU), alkaline phosphatase (ALP), creatine phosphokinase (CK), serum alanine aminotransferase (ALT), and aspartate aminotransferase (AST).

### 2.11. Bone Marrow Smear

Immediately following euthanasia, the sternum was dissected to collect bone marrow for smear preparation. The smears were stained with Wright–Giemsa stain and examined under a light microscope (× 40) to quantify metamyelocytes and orthochromatic erythroblasts [17].

### 2.12. Flow Cytometry

Under sterile conditions, approximately 200 mg of the spleen tail was surgically extracted, placed in a filter, and immersed in sterile PBS buffer. The spleen was gently pressed with a 5‐mL sterile syringe plunger to generate a suspension of spleen cells (SpC) [18]. Flow cytometry was employed to assess immune‐related parameters in the SpC suspension, particularly the expression levels of CD45^+^, CD3^+^, CD4^+^, CD8^+^, CD25^+^, CD161^+^, and CD11b/c^+^.

### 2.13. Gross Assessment and Histopathology

All animals underwent a comprehensive gross necropsy, which included a thorough examination of the external surfaces, orifices, skull, thorax, and abdominal cavity along with its contents. Internal organs and tissues were subsequently removed, cleaned of adhering tissues, blotted dry from saline, and weighed to obtain both absolute and relative weights. Target organs, including the heart, liver, kidneys, spleen, lungs, brain, and stomach, were fixed in 10% neutral buffered formalin for histopathological examination. Slides were prepared using an RM2235 rotary microtome (Leica, Germany) and examined with a Mias‐2000 pathological image processing system (China). The histopathological evaluation was performed by blinded study pathologists, with toxicity lesion severity graded (Table 1) using an incremental semiquantitative scoring system (0–4).

**Table 1:** Severity scores of the histopathological changes.

Score	Description
0	None
1	Minimal
2	Mild
3	Moderate
4	Severe

### 2.14. Statistical Analysis

All data were analyzed using the R programming language. Descriptive statistics were employed to assess normality. Independent samples *t*‐tests were conducted on toxicity data that met normal distribution criteria. For toxicity data that were not normally distributed, nonparametric Kruskal–Wallis tests and Mann–Whitney *U* tests were utilized, as appropriate, to compare means across replicate experiments. Parametric data are reported as mean ± standard deviation (SD), whereas nonparametric data are expressed as median and interquartile range (IQR). A significance level of *p* < 0.05 was adopted.

## 3. Results

### 3.1. Phytochemical Screening of Plant Extracts

To identify the phytochemical components present in the extract, a preliminary phytochemical analysis was conducted. The results confirmed the presence of polysaccharide bioactive components in the 4.7‐g crude drug per milliliter AWE suspension, with a total polysaccharide content of 131.3 mg/mL.

### 3.2. Morbidity and Mortality of Rats

Throughout the 90‐day administration period of the AWE, no treatment‐related morbidity or mortality was observed at the tested dose levels in rats.

### 3.3. General Observations and Behavioral Changes

All treated rats appeared normal during the treatment period. No abnormalities were observed in skin, fur, eye color, nasal discharge, feces, motor progression, or respiration.

### 3.4. Body Weight

During the 90‐day treatment period, all rats exhibited normal weight gain, and the weight gain patterns were not comparable between groups (*p* > 0.05) (Figure 1).

Figure 1Effects on body weight and weight gain in the repeated‐dose toxicity study.

**Figure:**
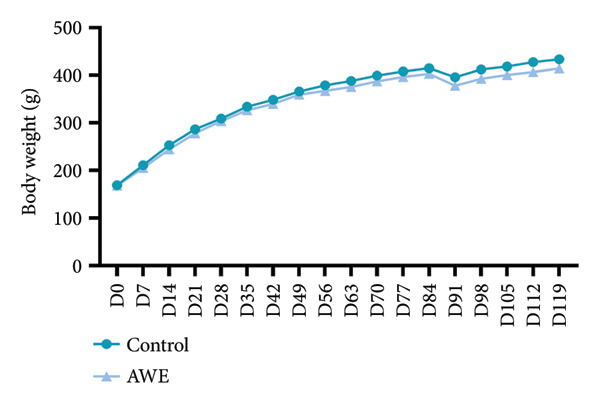
(a)

**Figure:**
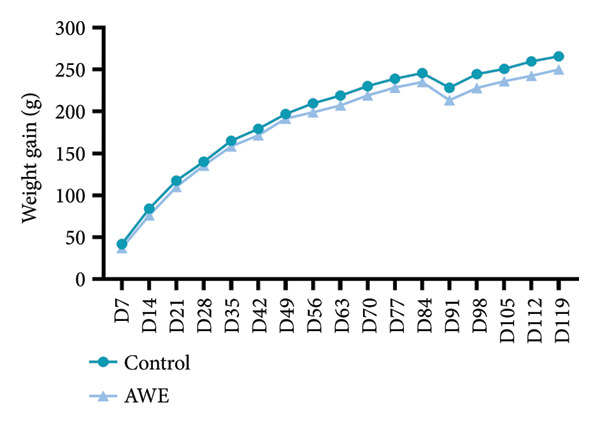
(b)

Data are presented as mean and were analyzed using independent samples *t*‐tests (D0‐D84 *n* = 30 animals/group, D91‐D119 *n* = 10 animals/group). No significant changes in body weight or weight gain were observed after 90 consecutive days of AWE administration (*p* > 0.05) nor were there any delayed toxic reactions.

### 3.5. Food Consumption

**Figure 2:**
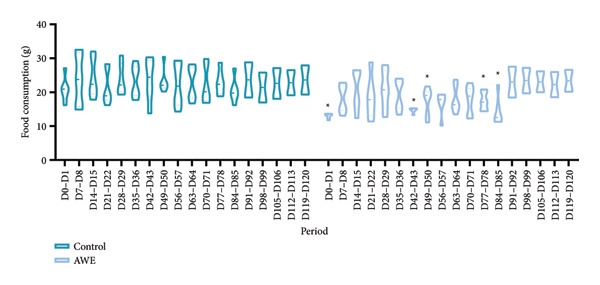
Effects on food consumption in the repeated‐dose toxicity study.

Food consumption was monitored throughout the experiment (Figure 2). The AWE group exhibited significantly reduced food consumption compared to the control group at the following time points: D0‐D1, D42‐D43, D49‐D50, D77‐D78, and D84‐D85 (*p* < 0.05). No significant differences were observed at other time points.

Data are presented as median and IQR and were analyzed using nonparametric Kruskal–Wallis and Mann–Whitney *U* tests (D0–D85 *n* = 30 animals/group, D91–D120 *n* = 10 animals/group). After 90 consecutive days of AWE administration, food consumption was significantly reduced at five time points (*p* < 0.05).

### 3.6. Changes in Rectal Temperature of Rats

**Figure 3:**
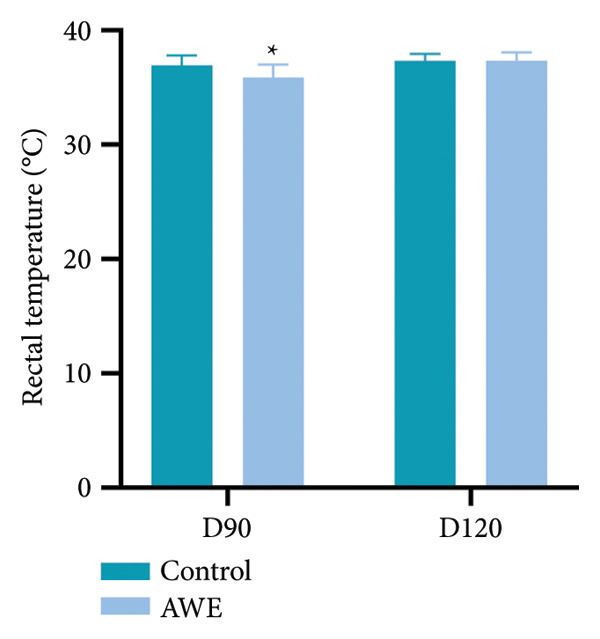
Changes in rectal temperature of rats.

Rectal temperatures were measured on Days 90 (D90) and 120 (D120) for all surviving rats (Figure 3). Compared to the control group, the AWE group exhibited a significant decrease in rectal temperature on D90 (*p* < 0.05).

Data are presented as mean ± SD and were analyzed using independent samples *t*‐tests (D90 *n* = 30 animals/group and D120 *n* = 10 animals/group). Compared to the control group, the AWE group showed a significant decrease in rectal temperature on D90 (*p* < 0.05), with no significant difference on D120 (*p* > 0.05).

### 3.7. AWE Shortened Latency Time in Rotarod Test and Recovered in 30 Days

**Figure 4:**
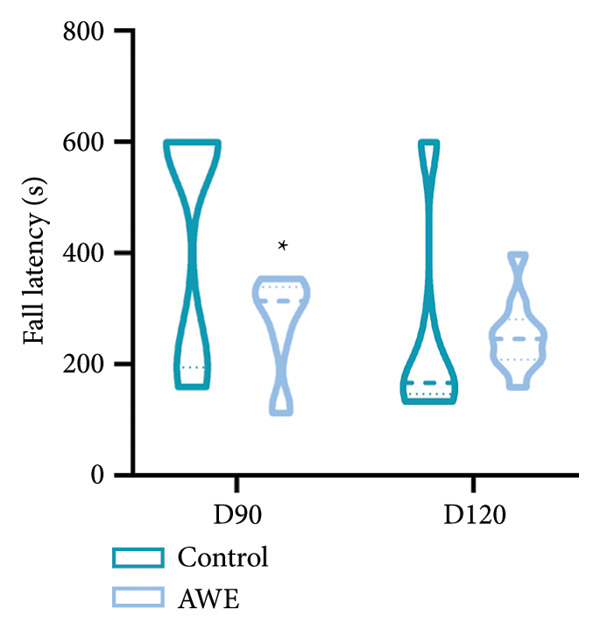
Changes in latency time in the rotarod test.

All surviving rats were assessed using the rotarod apparatus on Days 90 and 120 (Figure 4). The AWE group exhibited a significant reduction in latency time on Day 90 (*p* < 0.05). Compared to the control group, which returned to baseline levels after a 30‐day recovery period (*p* > 0.05).

Data are presented as median and IQR and were analyzed using nonparametric Kruskal–Wallis and Mann–Whitney *U* tests (D90 *n* = 30 animals/group and D120 *n* = 10 animals/group). Compared to the control group, the AWE group showed a significant shortening of the latency time on D90 (*p* < 0.05), with no significant difference on D120 (*p* > 0.05).

### 3.8. Clinical Hematology

After 90 days of AWE treatment, no significant (*p* > 0.05) effects were observed on any hematological parameters in the animals (Table 2).

**Table 2:** Hematological parameter after 90‐day AWE administration.

Categories	D91		D121	
	Control	AWE	Control	AWE
RBC (10^12^/L)	7.16 ± 0.46	6.98 ± 0.55	7.63 ± 0.74	7.54 ± 0.48
HGB (g/L)	144 ± 9	142 ± 7	144 ± 9	144 ± 7
MCV (fL)	53.1 ± 2.2	54.3 ± 2.7	52.2 ± 2.9	52.2 ± 2.0
MCH (pg)	20.1 ± 0.7	20.4 ± 1.0	19.0 ± 0.8	19.1 ± 0.6
MCHC (g/L)	379 ± 8	375 ± 9	364 ± 8	366 ± 5
WBC (10^9^/L)	3.66 ± 1.26	4.44 ± 1.47	4.19 ± 1.94	4.12 ± 1.85
PLT (10^9^/L)	860 ± 72	861 ± 85	911 ± 128	944 ± 97
PT (s)	15.2 ± 4.9	14.8 ± 4.3	12.4 ± 2.0	12.4 ± 1.2
APTT (s)	21.2 ± 10.2	20.1 ± 10.3	14.1 ± 2.0	14.4 ± 1.4

*Note*: Values are expressed as the mean ± SD. There was no significant (*p* < 0.05) difference between the control and AWE groups.

### 3.9. Clinical Biochemistry

After subacute oral administration of AWE to rats, no statistically significant differences (*p* > 0.05) were observed in any of the tested biochemical parameters (*p* > 0.05) (Table 3).

**Table 3:** Serum biochemistry parameters after 90‐day AWE administration.

Parameters	D91		D121	
	Control	AWE	Control	AWE
TP (g/L)	65.8 ± 6.5	64.5 ± 6.3	69.4 ± 10.5	63.2 ± 4.8
ALB (g/L)	36.5 ± 4.8	36.4 ± 4.4	38.3 ± 7.1	34.8 ± 4.3
CHOL (mmol/L)	1.82 ± 0.35	1.75 ± 0.35	2.00 ± 0.42	1.88 ± 0.30
TG (mmol/L)	0.533 ± 0.136	0.623 ± 0.191	0.521 ± 0.143	0.439 ± 0.149
GLU (mmol/L)	7.07 ± 0.57	7.13 ± 0.82	7.56 ± 1.31	7.14 ± 0.48
TBIL (μmol/L)	3.22 ± 0.71	2.81 ± 0.65	3.32 ± 1.45	2.77 ± 1.37
AST (IU/L)	122 ± 33	111 ± 29	144 ± 25	146 ± 48
ALT (IU/L)	34.3 ± 8.3	31.5 ± 6.7	47.7 ± 13.4	39.8 ± 8.1
ALP (IU/L)	87.7 ± 47.2	76.3 ± 34.3	63.6 ± 25.1	79.2 ± 32.6
CK (IU/L)	435 ± 245	428 ± 265	520 ± 134	587 ± 306
BUN (mmol/L)	4.36 ± 0.60	4.05 ± 0.50	4.90 ± 0.52	4.66 ± 0.57
CREA (μmol/L)	42.5 ± 5.3	42.0 ± 6.8	46.6 ± 4.3	43.4 ± 3.3
Potassium (mmol/L)	4.07 ± 0.27	4.09 ± 0.36	4.22 ± 0.43	4.18 ± 0.36
Sodium (mmol/L)	140.5 ± 1.6	139.7 ± 1.2	139.3 ± 0.5	139.5 ± 1.2
Chloride (mmol/L)	104.0 ± 1.3	103.3 ± 1.3	101.4 ± 1.4	102.0 ± 1.6

*Note*: Values are expressed as the mean ± SD. There was no significant (*p* < 0.05) difference between the control and AWE groups.

### 3.10. Changes in the Nuclear Cells of the Erythroid of the Bone Marrow

**Figure 5:**
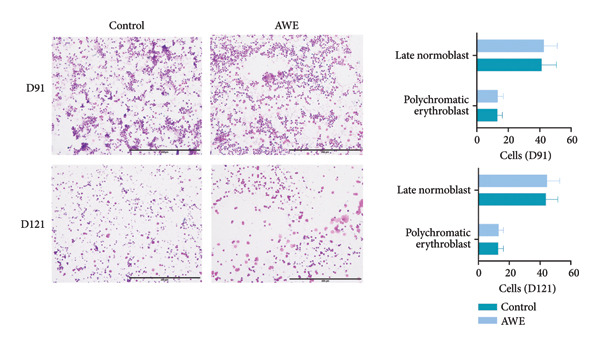
Microscopic examination of bone marrow staining smears of rats.

The investigation of bone marrow smears from the study animals revealed active megakaryocytopoiesis characterized by normal nuclear budding. Furthermore, no significant differences (*p* > 0.05) were observed in the counts of metamyelocytes and orthochromatic erythroblasts between the control and AWE groups (Figure 5).

In Wright–Giemsa composite stain, megakaryocytopoiesis is active, and nuclear budding is normal (× 40). Data are presented as mean ± SD and were analyzed using independent samples *t*‐tests (D91 *n* = 20 animals/group and D121 *n* = 10 animals/group). No significant changes were observed in the number of metamyelocytes and orthochromatic erythroblasts between the control and AWE groups on D91 and D121 (*p* > 0.05).

### 3.11. Changes in the Immune‐Related Parameters of SpC

**Figure 6:**
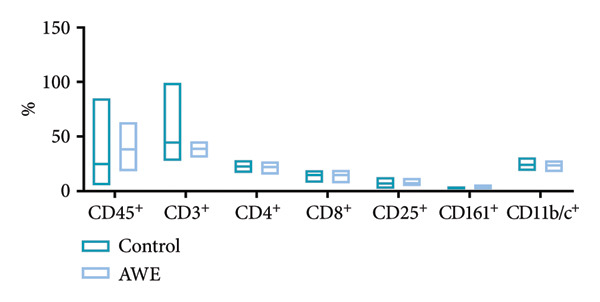
Flow cytometry examination of spleen cell suspensions at the end of dosing (D91).

Flow cytometry analysis of SpC from both the control and AWE groups at the conclusion of dosing (D91) demonstrated appropriate proportions of various cell types across all rats, with no significant alterations in spleen immune function (*p* > 0.05) (Figure 6).

Data are presented as median and IQR and were analyzed using nonparametric Kruskal–Wallis and Mann–Whitney *U* tests (*n* = 10 animals/group). No significant changes were observed in the immune‐related parameters between the control and AWE groups (*p* > 0.05).

### 3.12. Gross Examination

Gross necropsy revealed no treatment‐related gross findings in any of the rats. There were no observable changes in the external surfaces, orifices, skull, thorax, abdominal cavity, or their contents between the control and treated animals. All internal organs, including the heart, liver, kidneys, spleen, lungs, brain, and stomach, appeared healthy and normal in terms of shape, size, position, and color. No significant morphological or hemorrhagic changes were noted in these organs as a result of AWE.

### 3.13. Absolute and Relative Organ Weight

There were no significant differences (*p* > 0.05) in absolute or relative organ weights between the control and AWE groups (Tables 4 and 5).

**Table 4:** Absolute organ weights (g) of rats.

Parameters	D91		D121	
	Control	AWE	Control	AWE
Heart	1.416 ± 0.138	1.470 ± 0.086	0.912 ± 0.064	0.912 ± 0.131
Liver	13.73 ± 2.39	16.01 ± 1.74	8.27 ± 0.86	8.94 ± 1.20
Spleen	0.844 ± 0.131	0.770 ± 0.075	0.600 ± 0.078	0.635 ± 0.093
Kidney	3.40 ± 0.39	3.79 ± 0.35	2.10 ± 0.23	2.31 ± 0.26
Brain	2.22 ± 0.10	2.18 ± 0.08	2.02 ± 0.08	1.97 ± 0.13
Thymus	0.340 ± 0.061	0.579 ± 0.681	0.294 ± 0.061	0.300 ± 0.046
Adrenals	0.055 ± 0.012	0.051 ± 0.008	0.068 ± 0.008	0.065 ± 0.012
Fat	17.604 ± 5.994	18.068 ± 4.701	11.777 ± 3.008	9.236 ± 3.473
Brown fat	0.67 ± 0.19	0.64 ± 0.14	0.425 ± 0.102	0.498 ± 0.127

*Note*: Values are expressed as the mean ± SD. There was no significant (*p* < 0.05) difference between the control and AWE groups.

**Table 5:** Relative organ weights (g/100 g body weight) of rats.

Parameters	D91		D121	
	Control	AWE	Control	AWE
Heart	0.277 ± 0.028	0.286 ± 0.019	0.299 ± 0.022	0.312 ± 0.031
Liver	2.67 ± 0.27	3.10 ± 0.23	2.70 ± 0.18	3.06 ± 0.19
Spleen	0.166 ± 0.032	0.150 ± 0.015	0.197 ± 0.022	0.219 ± 0.033
Kidney	0.665 ± 0.077	0.737 ± 0.053	0.687 ± 0.073	0.793 ± 0.058
Brain	0.436 ± 0.043	0.425 ± 0.028	0.663 ± 0.038	0.679 ± 0.056
Thymus	0.067 ± 0.015	0.110 ± 0.121	0.097 ± 0.021	0.104 ± 0.019
Adrenals	0.011 ± 0.002	0.010 ± 0.001	0.022 ± 0.002	0.022 ± 0.005

*Note*: Values are expressed as the mean ± SD. There was no significant (*p* < 0.05) difference between the control and AWE groups.

### 3.14. Histopathology of Organs

**Figure 7:**
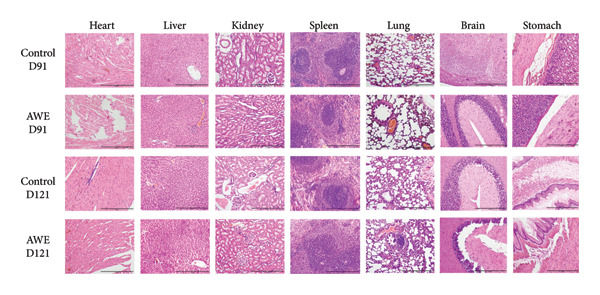
Histopathological examination of target organs. Scale bar: 200 μm.

Histopathological examination of target organs, including the heart, liver, kidneys, spleen, lungs, brain, and stomach, revealed no significant abnormalities associated with subacute treatment using AWE (Figure 7).

## 4. Discussion

Herbal and traditional medicines are extensively utilized in developing countries and are classified as complementary and alternative medicine (CAM) in the Western world [19]. These medicines are frequently employed for the prevention and treatment of various diseases, as well as for promoting overall health. In the past, a significant portion of the global population experienced acute COVID‐19 infections, with over 10% of patients developing long COVID or postacute sequelae of SARS‐CoV‐2 infection (PASC) [20]. The most prevalent symptoms include fatigue (53.1%), shortness of breath (43.4%), joint pain (27.3%), and chest pain (21.7%) [21]. Joung et al. conducted a prospective observational study involving 50 patients and found that the administration of *A. membranaceus* significantly improved fatigue symptoms in individuals suffering from long COVID [22]. The alleviation of fatigue associated with stroke and cancer is a primary reason for the increased consumption of *A. membranaceus* in hospital settings [23, 24]. Furthermore, elderly individuals are often more susceptible to fatigue, which further drives the consumption of *A. membranaceus* within this demographic as China’s population ages [25]. Outside of hospital settings, *A. membranaceus* is typically self‐prepared and consumed by individuals, with doses rarely monitored. Many individuals take it for prolonged periods, believing these medications to be free from side effects or adverse reactions. Therefore, well‐designed scientific studies are essential for this category of herbal and traditional medicines.

Documented evidence suggests that *A. membranaceus* demonstrates favorable safety profiles at lower repeated dosing levels. Given the extensive historical use of *A. membranaceus* spanning over 2000 years, along with the simulation of traditional clinical practices in its extraction, we employed a high‐dose repeated‐dose administration approach to systematically evaluate the toxicity of AWE in Sprague–Dawley rats. According to the Chinese Pharmacopoeia, the oral dose of *A. membranaceus* ranges from 9 to 30 g of crude drug per day, which translates to a maximum daily dose of 0.5 g of crude drug per kilogram for an adult, assuming a body weight of 60 kg. In our study, the dose of AWE administered was 94 times the maximum daily dose recommended for adults. During the study period, several statistically significant changes were observed, including reduced food intake during the dosing period, decreased rectal temperature at the end of dosing, and decreased physical activity (Figures 2, 3 and 4). We consider that these findings, although statistically significant, are likely to be of no toxicological relevance.

In evaluating the toxicological relevance of the findings from toxicity tests, it is customary to consider both the extent of organ damage and its reversibility [26].

Results related to body weight and fat content indicated that changes in the AWE group were not statistically significant (Figure 1 and Table 4), suggesting that the impact of high‐dose AWE consumption on growth was minimal.

Gastrointestinal discomfort is a common adverse effect of medications [27]. The observed reduction in food consumption in rats suggests that repeated high‐dose intake of AWE may have induced gastrointestinal discomfort. A decrease in food intake implies a corresponding reduction in energy and protein consumption. Protein intake is known to effectively elevate body temperature and energy expenditure [15], while reduced energy intake can adversely affect thermoregulation and decrease physical activity capacity [28]. Notably, the reduction in food consumption was a significant factor contributing to the decreased rectal temperature and shortened latency to fall in the rotarod test (Figures 3 and 4). Furthermore, parameters from clinical pathology examinations did not indicate severe organ damage (Tables 4 and 5, Figure 7) in the AWE group. Therefore, high‐dose consumption of AWE did not exhibit significant toxic effects overall.

## 5. Conclusion

Although high‐dose AWE administration induced transient changes in food consumption, body temperature, and decreased physical activity, these effects were reversible during the recovery period and not associated with major organ toxicity. These findings support the relative safety of AWE at high doses, although further studies are warranted to define safe exposure limits in humans.

## Conflicts of Interest

The authors declare no conflicts of interest.

## Author Contributions

Wei Du and Ping Zhang contributed equally to this work.

## Funding

This research has received funding from China’s Key Research and Development Program (No. 2022YFC3501603).

## Data Availability

The data used to support the findings of this study are available from the corresponding authors upon reasonable request.
